# Risk of Proton Pump Inhibitor (PPI)-Induced Galactorrhea: An Uncommon Adverse Effect of a Common Drug

**DOI:** 10.7759/cureus.81050

**Published:** 2025-03-23

**Authors:** Fezaan A Sheikh, Zamanali Khakhar, Gagandeep Bhogal, Alok Iyer, Sayed K Ali

**Affiliations:** 1 Medicine, University of Western Australia, Perth, AUS; 2 Medicine, University of Nairobi, Nairobi, KEN; 3 Medicine, Aga Khan University Hospital, Nairobi, KEN

**Keywords:** chronic kidney disease, galactorrhea, gastroesophageal reflux disease (gerd), prolactin, proton-pump inhibitors (ppi), rabeprazole

## Abstract

Galactorrhea is a rare and atypical adverse effect associated with the use of proton pump inhibitors (PPIs). They are widely used in the management of acid-related disorders and have been proven to be effective. However, like all pharmacological therapies, PPI use is accompanied by a range of adverse effects. This case report details a 27-year-old female individual with no significant medical history who presented with galactorrhea. Upon further investigation, PPI-induced galactorrhea was confirmed and discontinuation of the medication resulted in the resolution of the condition. Complex interactions between the drug, various enzymes, and receptors have been postulated as potential mechanisms underlying the development of this condition. Chronic kidney disease has also been shown to augment the development of PPI-induced galactorrhea. Further research around the mechanism driving PPI-induced galactorrhea in patients is necessary to better enable healthcare professionals to provide accurate information concerning the side effect profile of PPIs and educate patients on informed decision-making regarding PPI therapy.

## Introduction

Prolactin, also known as lactotropin or mammotropin, is a polypeptide secreted by the anterior pituitary. It promotes growth of mammary glands and stimulates milk production. Dysfunctional secretion of prolactin can precipitate galactorrhea which manifests as milky discharge from the nipple unrelated to the physiological milk production in pregnant or breastfeeding women. Iatrogenic hyperprolactinemia can be associated with medications such as antipsychotics, metoclopramide, and antidepressants, which exert their effect through dopamine antagonism [1].

Proton pump inhibitors (PPIs) are commonly used as an effective treatment for gastroesophageal reflux disease (GERD) and other acid-related disorders. PPIs act to decrease gastric acid secretions by irreversibly binding to the H+/K+ ATPase, otherwise known as the proton pump, of gastric parietal cells. The combination of a favorable safety profile and high therapeutic efficacy in the management of dyspepsia renders PPIs a desirable long-term treatment, thereby contributing to their extensive use [2]. They are generally well tolerated, with headache, diarrhea, nausea, and vomiting being the most commonly reported adverse effects, occurring in less than 5% of users [3].

Despite this, there is growing concern over the potential for rare, more serious adverse effects associated with PPI usage including fractures, pneumonia, enteric infections, and acute interstitial nephritis [4]. While these complications are rare, the widespread utilization of PPIs allows rare effects to be unmasked. This case report seeks to elucidate the rare presentation of galactorrhea associated with the use of PPI (rabeprazole) in a 27-year-old, non-pregnant female individual receiving PPI therapy for the management of epigastric pain and dyspeptic symptoms.

## Case presentation

A 27-year-old female individual with no significant past medical history, presented with a three-day history of localized, non-radiating, mid-epigastric pain, exacerbated by food intake. The pain was accompanied by a sour taste in the mouth, most pronounced in the mornings. She did not report any associated symptoms such as fever, unintentional weight loss, or melena. Her physical exam was normal except for mid-epigastric tenderness on deep palpation. Her laboratory work included a urinalysis, complete blood count, liver function test, stool *Helicobacter pylori*, and lipase, which were all within normal limits. A diagnosis of gastro-esophageal reflux disease (GERD) was made and she was prescribed rabeprazole 20 mg once a day. 

After 10 days of treatment, she came back to the clinic complaining of sudden onset bilateral galactorrhea. Upon further evaluation, she did not report any associated symptoms such as headache or visual disturbance. She also denied concurrent use of other medications including oral contraceptives, antiemetics, H2-receptor antagonists, dopamine receptor antagonists, antipsychotics, or illicit substances. Her physical examination was otherwise normal, including a normal visual assessment, except for the presence of bilateral milk discharge upon manual expression.

The patient underwent further diagnostic workup which involved a complete blood count, electrolyte levels, liver function test, thyroid function test, renal function test, prolactin levels, and serum beta-human chorionic gonadotropin (β-hCG) (Table 1). A pregnancy test was negative.

**Table 1:** Patient laboratory results. BUN, blood urea nitrogen; GGT: gamma-glutamyl transferase; AST: aspartate aminotransferase; ALT: alanine aminotransferase; TSH: thyroid stimulating hormone; β-hCG: beta-human chorionic gonadotropin.

Tests	Results	Normal range
Hemoglobin	15.2 g/dl	12 - 17.5 g/dl
Hematocrit	44.60%	41 - 50%
White blood cell count	5.0 × 10^9^/L	4.5 - 11.0 × 10^9^/L
Platelets	373 × 10^9^/L	150 - 400 × 10^9^/L
Sodium	137.10 mmol/L	136 - 145 mmol/L
Potassium	4.51 mmol/L	3.5 - 5.1 mmol/L
Creatinine	54.6 µmol/L	62 - 115 µmol/L
BUN	2.20 mmol/L	3.2 - 8.2 mmol/L
GGT	11.20 U/L	0.00 - 38 U/L
AST	21.70 U/L	0.00 - 34 U/L
ALT	12.30 U/L	0.00 - 49 U/L
Alkaline phosphatase	88.10 U/L	46 - 116 U/L
TSH	0.755 µIU/ml	0.30 - 3.94 µIU/ml
Free T4	1.15 ng/dl	0.95 - 1.57 ng/dl
Free T3	3.13 pg/ml	2.42 - 4.36 pg/ml
Prolactin	245 ng/mL	4.79 - 23.3 ng/mL
Serum β-hCG	< 20 IU/L	< 5 IU/L

A bilateral breast ultrasound demonstrated dilated retroareolar ducts containing anechoic fluid. No intraductal masses were identified in either breast. 

Based on the patient's normal neurological examination and existing financial constraints, a brain MRI was temporarily deferred. The patient was advised to discontinue rabeprazole, and a repeat prolactin level assessment after 10 days had returned to normal; 15.0 ng/mL. Using the World Health Organization-Uppsala Monitoring Centre (WHO-UMC) causality assessment, the association between rabeprazole use and the occurrence of galactorrhea can be classified as probable/likely [5]. The resolution of symptoms following discontinuation of the drug supports this causality assessment.

## Discussion

The literature on PPI-induced galactorrhea remains scarce, with only a few isolated reports available that primarily describe its occurrence in patients with end-stage chronic kidney disease. It is likely that PPIs are interacting with the regulatory pathways for prolactin secretion by either stimulating its secretion or inhibiting its suppression.

As this phenomenon is more commonly reported in those with kidney disease, one hypothesis suggests PPIs impair renal prolactin clearance resulting in hyperprolactinemia and subsequent galactorrhea. A cohort study found that PPI usage was associated with incident acute kidney injury and chronic kidney disease, thus supporting this theory [6].

PPIs may have a direct effect on the lactotrophs of the anterior pituitary. Computational modeling suggests that PPIs form stable complexes with D2 receptors on lactotrophs, potentially acting as D2 receptor antagonists, thereby blocking dopamine's inhibitory effect on prolactin release [7].

Another hypothesis is based on the potential for PPIs to increase estrogen levels hence inducing hyperprolactinemia and galactorrhea [8,9]. Omeprazole and esomeprazole are hepatically metabolized by cytochrome P450 2C19 (CYP2C19) and cytochrome P450 3A4 (CYP3A4) with the majority of metabolites then renally excreted [10]. Importantly, 5-O-desmethylomeprazole, a metabolite common to both omeprazole and esomeprazole, is a time-dependent inhibitor of CYP3A4 [11]. Since estrogen is metabolized into inactive forms by CYP3A4, its inhibition may lead to increased estrogen levels. Higher estrogen levels stimulate the release of prolactin from the anterior pituitary, as seen in pregnancy. However, high estrogen levels inhibit the effects of prolactin on mammary glands suggesting galactorrhea will only present with moderate inhibition of estrogen metabolism [12]. This proposed mechanism depends on the build-up of 5-O-desmethylomeprazole as an inhibitory metabolite to CYP3A4. In patients with kidney disease, poor renal clearance will cause this build-up. However, in patients with normal kidney function, an alternative explanation may be necessary. In these patients, genetic polymorphisms in CYP genes might predispose to poor estrogen metabolism, even at low concentrations of inhibitory metabolites. Genetic profiling of CYP genes in these patients may generate a better understanding of the mechanisms behind PPI-induced galactorrhea [13].

Galactorrhea is an uncommon side effect of PPIs. With the increased usage and availability of PPIs, these rare side effects may become more apparent. In this case, galactorrhea was confirmed and the condition resolved within a few days following discontinuation of the drug. It is essential to rule out brain tumors as a potential cause of hyperprolactinemia in patients presenting with galactorrhea. Based on the multiple hypothesized mechanisms of PPI-induced galactorrhea, there is a need for larger observational studies and mechanistic investigation to better understand this association and help guide clinical practice.

## Conclusions

Healthcare workers should be aware of these rare side effects in order to educate patients and facilitate informed decision making regarding PPI therapy. Further research around the mechanism driving PPI-induced galactorrhea in patients is necessary to better enable healthcare professionals to provide accurate information concerning the side effect profile of PPIs and potentially improve patient care by minimizing the development of unwanted effects of commonly used drugs.
